# Rapid Diagnosis of Rhabdomyolysis with Point-of-Care Ultrasound

**DOI:** 10.5811/westjem.2016.8.31255

**Published:** 2016-11-02

**Authors:** Alicia Nassar, Richard Talbot, Ashley Grant, Charlotte Derr

**Affiliations:** * University of South Florida, Morsani College of Medicine, Tampa, Florida; † University of South Florida, Division of Emergency Medicine, Tampa, Florida

## Abstract

It is important to rapidly diagnosis and treat rhabdomyolysis in order to decrease morbidity and mortality. To date there are no reports in the emergency medicine literature on the use of point-of-care ultrasound in the diagnosis of rhabdomyolysis. This unique case describes how ultrasound was used in the emergency department (ED) to quickly diagnose and treat rhabdomyolysis prior to confirmation with an elevated serum creatine kinase. When coupled with a high index of suspicion, ultrasound can be one of the most portable, readily available, low cost, and minimally invasive techniques for making a rapid diagnosis of rhabdomyolysis in the ED.

## INTRODUCTION

Rhabdomyolysis is the breakdown of skeletal muscle that can rapidly progress to acute renal failure or death. It is important to make a rapid diagnosis and initiate treatment for rhabdomyolysis in order to decrease morbidity and mortality. To date there are no reports in the emergency medicine literature on the use of point-of-care ultrasound in the diagnosis of rhabdomyolysis. This unique case describes a patient who presented to the emergency department (ED) with localized musculoskeletal pain. Using ultrasound, the patient was quickly diagnosed and treated for rhabdomyolysis prior to confirmation with an elevated serum creatine phosphokinase (CPK). When coupled with a high index of suspicion, ultrasound can be one of the most portable, readily available, low-cost, and minimally invasive techniques for making a rapid diagnosis of rhabdomyolysis in the ED.

## CASE REPORT

A 24-year-old male presented to the ED with a two-day history of bilateral arm pain. The pain was constant, located primarily to the biceps region of his upper arms. His pain began shortly after weight lifting. Past medical history included bipolar disorder and polysubstance abuse, including recent use of cocaine, marijuana, and a synthetic marijuana known as “spice.”

On arrival to the ED the patient had a temperature of 97.9 F, blood pressure of 167/88, heart rate of 122, and respirations of 16 per minute. Physical findings included bilateral biceps swelling and fullness with diffuse tenderness to the musculature. There was no external evidence of trauma. The patient’s upper extremities were neurovascularly intact with no evidence of paresthesias, weakness, or pallor. He was noted to display paranoid behavior without features of acute psychosis.

**Figure 1:**
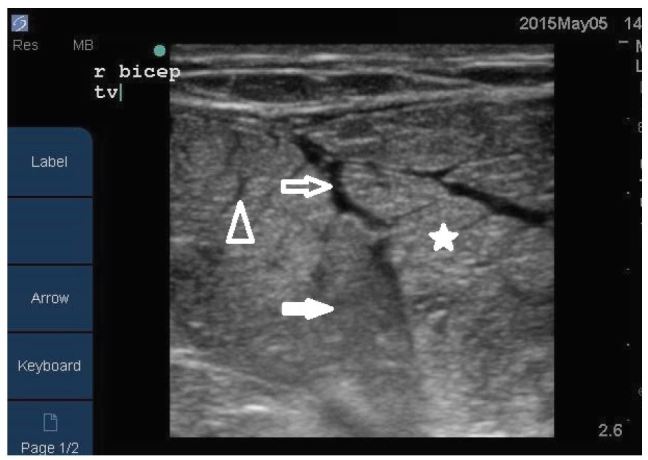
Transverse image of rhabdomyolysis of the right biceps muscles using a linear array transducer. Areas of increased and decreased echogenicity are seen, as well as disorganized muscle fibers within surroundings areas of fluid. The arrowhead is pointing towards the disorganized muscle fibers. The open arrow is pointing to areas of fluid. The closed arrow is pointing to areas of decreased echogenicity. The star is indicating the areas of hyperechogenicity.

A point-of-care musculoskeletal ultrasound was performed by the emergency physician to evaluate for a possible muscle or biceps-tendon tear. The sonogram showed areas of both increased and decreased echogenicity of the biceps muscle, as well as disorganized muscle fibers with surroundings areas of fluid (Figure 1, Video 1). There was preservation of the muscle boundary and the biceps tendon was intact. A presumptive diagnosis of rhabdomyolysis was made pending laboratory testing and the patient was started on intravenous (IV) fluids.

The patient’s lab work in the ED was notable for a creatine phosphokinase (CPK) of 83,000 U/L. AST/ALT were 813/169 IU/L respectively, total bilirubin of 1.5 mg/dl, and bicarbonate of 16 mEq/L. Urine results included amber color and “large” hemoglobin with 3–5 red blood cells per high powered field. A complete blood count, complete metabolic profile, and urinalysis were otherwise unremarkable. A urine drug screen was positive for marijuana.

The patient was hospitalized and treated with aggressive IV fluids. His CPK peaked at 124,000 and subsequently improved daily thereafter. His liver enzymes improved as well. A hepatitis panel led to a new diagnosis of hepatitis C, though liver enzyme elevation was thought to be primarily related to his acute rhabdomyolysis. Renal function remained normal throughout his stay. His hospital course was complicated by paranoia, psychosis, and aggression toward hospital staff. He was subsequently placed under an involuntary psychiatric hold and required security intervention and sedation. He was transferred to the local mental health facility on hospital day five for further psychiatric care after his rhabdomyolysis resolved.

## DISCUSSION

Rhabdomyolysis is a syndrome of skeletal injury that can rapidly progress to acute renal failure or even death.^1^,^2^ It is imperative that it be diagnosed and treated appropriately to reduce the lasting effects of the disease.^1^ Rhabdomyolysis results from a myriad of conditions including trauma, illicit drugs, medications, infection, excessive exercise, immobilization and psychiatric condition.^2^ The symptoms of rhabdomyolysis are as variable as the causes: from a patient with a traumatic crush injury, who has pigmented urine and renal failure, to one with no significant history of trauma who may simply present with fatigue, nausea, fever or muscle weakness.^1^,^2^ For these reasons, diagnosing rhabdomyolysis cannot be based on history and physical alone and generally requires a serum CPK that is greater than five times the normal limit, without evidence of brain or cardiac injury. 3

Once a diagnosis of rhabdomyolysis is suspected, additional workup is needed. An electrocardiogram should be performed to screen for conduction abnormalities caused by hyperkalemia including: peaked T waves, prolonged PR interval and a widened QRS complex.^2^ A metabolic panel should be performed to assess renal function, calcium, potassium and phosphorous levels.^1^ CBC and coagulation studies should be done to monitor for evidence of DIC.^1^

**Figure 2:**
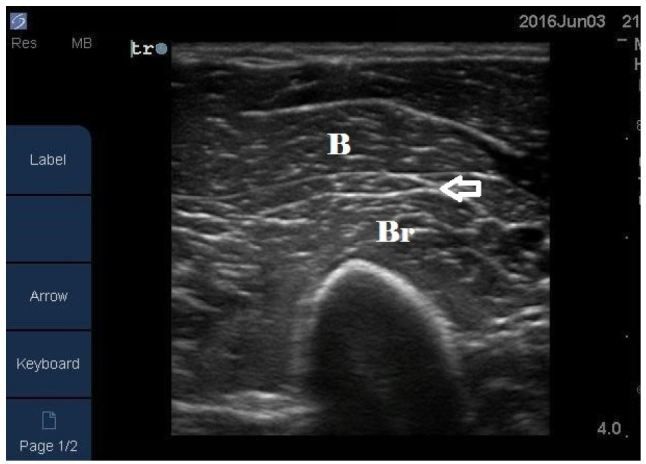
Transverse image of a normal left biceps muscle (B) and brachialis muscle (Br) using a linear array transducer. Normal skeletal muscle has a relatively hypoechoic echotexture with clearly demarcated linear hyperechoic strands of fibroadipose septa (arrow).

Imaging may be required in patients who present with localized pain. Ultrasound and magnetic resonance imaging (MRI) can both be used to determine the extent of muscular damage that exists. On ultrasound normal skeletal muscle has a relatively hypoechoic echotexture with clearly demarcated linear hyperechoic strands of fibroadipose septa^4^ (Figure 2). In rhabdomyolysis the findings are variable but may include hyperechoic areas of muscle,^5^,^6^ which is likely due to hypercontractile muscle fibers in the acute phase of muscle injury,^4^ hypoechoic areas of muscle,^4^,^7^ which is believed to be caused by edema and inflammation of the muscle,^4^,^7^ increased muscle thickness, and fluid within the surrounding the muscles.^4^ Areas of locally disorganized fascicular architecture^8^ may also be appreciated but generally the muscle boundary itself remains intact unless an associated tear is present.^8^ The area of locally disorganized fasicular architecture is thought to represent necrosis of the muscle.^4^,^7^

Abscesses and hematomas may also appear anechoic and hypoechoic and should be considered on the differential diagnosis.^5^ However, on examination neither of these conditions correlates with the clinical picture. With both an abscess and hematoma one would expect a localized fluid collection on ultrasound in conjunction with physical exam findings of infection and trauma respectively. Additionally, there would not be a significantly elevated serum CPK or increase in creatinine level associated with these diagnoses.

Clinicians performing soft tissue ultrasound should be familiar with the sonographic changes associated with other common musculoskeletal pathologies such as muscle tears, strains, and contusions in order to avoid diagnostic error. Complete muscle tears are associated with avulsion and retraction of the injured muscle segment along with an adjacent anechoic or hypoechoic hematoma.^9^ Partial tears, strains, and contusions have focal hypoechoic areas within the muscle fibers themselves, representing localized edema and hemorrhagic changes that have disrupted the normal fibroadipose pattern.^9^ Unlike in rhabdomyolysis, the sonographic findings seen with tears, strain injuries, and contusions are limited to a focal area of injury and are not diffusely present throughout the involved muscle. MRI is excellent at demonstrating rhabdomyolysis with T2-weighted MRI images of rhabdomyolysis generally show increased signal intensity.^8^ Unfortunately performing an MRI is associated with greater cost and long testing times. In addition, MRI is not readily available in most EDs.

The initial treatment of rhabdomyolysis involves early and aggressive IV fluid resuscitation.^2^ The patient should be admitted and monitored for life-threatening complications such as acute renal failure and hyperkalemia.

In this case, the patient presented with bilateral upper extremity pain and swelling and the ultrasound was used to narrow the differential of possible musculoskeletal pathologies. A presumptive diagnosis of rhabdomyolysis was made and later confirmed by serum CPK levels. If a patient has sonographic findings suggestive of rhabdomyolysis, treatment can be initiated immediately while laboratory testing is still in progress. The increasing availability of point-of-care ultrasound in most EDs makes it an ideal imaging modality for the initial evaluation of the patient in whom rhabdomyolysis is considered.

## LIMITATION

There are no follow-up ultrasounds available to show this patient’s return to normal muscles.

## Figures and Tables

**Video:** This ultrasound video is of the patient’s left bicep muscle. It shows disorganized muscle fibers with surrounding areas of anechoic fluid. The biceps muscle displays areas of both increased and decreased echogenicity. There is preservation of the muscle boundaries within the echogenic fascial planes.

## References

[1] Bagley WH, Yang H, Shah KH (2007). Rhabdomyolysis. Intern Emerg Med.

[2] Zimmerman JL, Shen MC (2013). Rhabdomyolysis. Chest.

[3] Reha WC, Mangano FA, Zeman RK (1989). Rhabdomyolysis: need for high index of suspicion. Urology.

[4] Chiu Y-N, Wang T-G, Hsu C-Y (2008). Sonographic Diagnosis of Rhabdomyolysis. J Med Ultrasound.

[5] Fornage BD, Nerot C (1986). Sonographic diagnosis of rhabdomyolysis. J Clin Ultrasound.

[6] Steeds RP, Alexander PJ, Muthusamy R (1999). Sonography in the diagnosis of rhabdomyolysis. J Clin Ultrasound.

[7] Kaplan GN (1980). Ultrasonic appearance of rhabdomyolysis. AJR Am J Roentgenol.

[8] Moratalla MB, Braun P, Fornas GM (2008). Importance of MRI in the diagnosis and treatment of rhabdomyolysis. Eur J Radiol.

[9] Bianchi S, Marinoli C (2007). Ultrasound of the Musculoskeletal System.

